# Supplementary material for: Anti-chikungunya virus seroprevalence in Indigenous groups in the São Francisco Valley, Brazil
Source: PLoS Negl Trop Dis. 2021 Jun 28;15(6):e0009468. doi: 10.1371/journal.pntd.0009468 (PMC8238182; doi:10.1371/journal.pntd.0009468)
Supplement: S1 Table — (DOCX) [file pntd.0009468.s003.docx]

|  | **Control (n=52)** | **Fulni-ô (n=272)** | **Truká (n=109)** |  |
| --- | --- | --- | --- | --- |
| **Variables** | **Median (1Q - 3Q)** | **Median (1Q - 3Q)** | **Median (1Q - 3Q)** | ***P*** |
| **Age, years** |  |  |  |  |
| Seropositive | 49·0 (41·0 - 59·0)** | 47·0 (37·0 - 54·0)^#^ | - | 0·528 |
| Seronegative | 47·0 (38·5 - 56·0)** | 47·5 (38·0 - 56·0)^#^ | 46·0 (39·0 - 53·0) | 0·663 |
| **Body mass index, kg/m^2^** |  |  |  |  |
| Seropositive | 30·1 (29·8 - 32·5)^†^ | 28·1 (25·2 - 30·7)^‡^ | - | 0·354 |
| Seronegative | 28·7 (24·9 - 31·8)^†^ | 26·2 (24·2 - 30·5)^‡^ | 29·1 (25·7 - 32·9) | 0·364 |

**S1 Table**. Analysis of median values of age and BMI with anti-chikungunya virus seroprevalence

LEGEND: 1Q – first quartile; 3Q – third quartile; *72 CHIKV IgG+ individuals with missing weight and/or height, all from the Fulni-ô group; **p = 0·757; ^#^p = 0·520; ^†^p = 0·340; ^‡^p = 0·797
