# Supplementary material for: Anti-chikungunya virus seroprevalence in Indigenous groups in the São Francisco Valley, Brazil
Source: PLoS Negl Trop Dis. 2021 Jun 28;15(6):e0009468. doi: 10.1371/journal.pntd.0009468 (PMC8238182; doi:10.1371/journal.pntd.0009468)
Supplement: Attachment — Submitted filename: Rebuttal LetterPLOS1 DEFINITIVOdocx.docx [file pntd.0009468.s004.docx]

**REBUTTAL LETTER**

On behalf of all authors, we thank the reviewers for the thorough comments and suggestions on our original manuscript. The points raised were extremely helpful to enhancing our work. Please find in this resubmission a restructured version of our study. In addition, we provide detailed answers to each of your concerns in this rebuttal letter, with correspondent modifications highlighted in the submitted manuscript. We appreciate the opportunity to resubmit our study and hope that the revised paper will be fit for publication in *PLOS NEGLECTED TROPICAL TROPICAL DISEASES*.

Sincerely,

Jandir Mendonça Nicacio, MD

Anderson Armstrong, MD, MSc, PhD

**Comments to the Author:**

The reviewers provided a number of issues to address to improve the manuscript, including:

- The Methods section needs to be clarified in a number of places, as mentioned below. For example, there should be more description of the PAI study and the sampling methodology.

***ANSWER:*** *Thanks for pointing it out. We now have added information in the Methods section (“***Study design and sample selection” and “Statistical Analysis”) –** Lines 45-61 and Lines 124-132

- The interpretation of the data needs to be better justified in some places, including the mention that this population is most endangered for new viral outbreaks. In addition, the data just seems surprising and the reasons for the outcome should be explored a bit more - the prevalence is extremely high in one relatively remote community, but zero or nearly zero in communities you'd expect to have higher exposure. Also, how many acute cases were reported from these regions? >78% attack rate is quite high - was there a recognized outbreak in recent years? Hard to imagine a 78% attack rate would've gone unnoticed, but there's no context provided here.

Other issues to address, in addition to the comments by the reviewers:

- The concept of a 'control' population is not clear in the manuscript. In what way would a more urbanized population be a control? Where these anticipated to be more highly positive? The hypothesis being tested that would make this population the control is not clear and should be explained

***ANSWER:*** *Thanks for pointing it out. We now have added information in the INTRODUCTION section* **–** Lines 38-43 and *METHODS* section (**Indigenous Groups)-** Lines 63-68.

*This study describes the serological and epidemiological profile of CHIKV in two Indigenous populations in the Northeast of Brazil, at different stages of urbanization, in addition to an urbanized control community in the same region. The city of Juazeiro was chosen for the control group, as it is a totally urbanized region, with similar climatic conditions, also located in the Northeast of Brazil, in the São Francisco Valley mesoregion.*

- Line 10: Mentions a million cases in Kenya and India, but the references don't actually speak to those numbers. Were there really a million cases in Kenya?

***ANSWER:*** *Thanks for pointing it out. We clarified this information in the INTRODUCTION section* **–** Lines 9,10

- Line 23: "CHKF”

***ANSWER:*** *Thanks for pointing it out. We clarified this information in the INTRODUCTION section* **–** Lines 23. “CHIKF”

- Lines 49-51, Ethical issues: The PAI study was approved by the various stakeholders, but was this add-on CHIKV study specifically approved?

***ANSWER:*** *Thanks for pointing it out. We now have added information*

*in the METHODS section* **–** Lines 57-61.

-Lines 58-63: A map might be helpful in explaining how these communities are situated relative to one another.

***ANSWER:*** *Thanks for pointing it out. We now have added information*

*in the METHODS section (***Indigenous Groups) –** Below the line 85.

- Methods: what was the timing of the sampling? Line 73 says there were two collection periods, but it wasn't clear which groups were sampled when.

***ANSWER:*** *Thanks for pointing it out. We now have added information*

*in the METHODS section (“***Anthropometric data collection” and “Collection of biological material”) – Lines: 89-95 and Line: 102**

**“**During EW 34/2016, only anthropometric data, and biological samples were collected in the Fulni-ô group.”

“In EW 22/2017, a second moment of collecting anthropometric data and biological samples was performed, now not only in the Fulni-ô group, but also in the Truká tribe and in the control group.”

“Peripheral blood collections were also performed in EW 22/2017…”

- Lines 89-90: How many samples were discarded? How did that impact overall participant numbers?
- ***ANSWER:*** *Thanks for pointing it out. We now have added information*

*in the METHODS section (“***Serological tests”) – Lines: 111-115**

- Lines 108-110: The wording in these sentences is odd. I don't usually see 'prevalence' represented first as a count number. I recommend referring to the proportion first, with the total number of positives in parentheses. E.g. "The prevalence of anti-CHIKV IgG in the study sample was 49.9% (216/433; 95% CI: 45.1 - 54.7)"
- ***ANSWER:*** *Thanks for pointing it out. We now have added information*

*in the RESULTS section***– Line: 150** “The prevalence of anti-CHIKV IgG was 49·9% (216/433; 95% CI: 45·1 – 54·7)”.

Lines 115-116: The comment that anti-CHIKV antibodies were more common in females than males (66.2% vs 33.8%) is very confusing - this is largely just a function of the fact that more females participated in the study. Along these same lines, on Table 2, it would be easier to interpret if the data were presented as the percent antibody positive rather than the percent of each category. That is, the number in the bracket should be the percentage of each group/variable that are positive or negative for anti-CHIKV antibodies.

***ANSWER:*** *Thanks for pointing it out. We clarified this information in the RESULTS section* **–** Lines: 150, 151 (the comment was withdrawn) and Table 2.

- Lines 128-130 and Table 3: I don't follow how the prevalence ratio calculation was approached. What was it adjusted for? How is Truka the reference category when the prevalence is zero? How should these numbers be interpreted?

***ANSWER:*** *Thanks for pointing it out. We clarified this information in the RESULTS section***.** *After reviewing the statistical data and the variables presented, we conclude that multivariate analysis is not appropriate. Therefore, we portrayed the misconception and agreed with the reviewer.*

Lines 136-138: This statement is a repeat from earlier in the Results.

***ANSWER:*** *Thanks for pointing it out. We clarified this information in the RESULTS section. This statment was removed from the manuscript.*

- Lines 167-175: This paragraph needs a great deal of additional clarification - the paragraph eitherneeds to be rewrittenor deleted entirely. How is "climate" involved here? What was the timing of the mosquito survey? What is a rainfall index and how does it relate to Aedes abundance? I would expect that all of these locations could harbor Aedes mosquitoes, so this paragraph should focus on explaining what mosquito surveys were done at all 3 locations and when, with what regularity, and how that might relate to understanding CHIKV transmission. Aedes populations can be quite dynamic, so a single survey might not really capture transmission potential.
- ***ANSWER:*** *Thanks for pointing it out. We now have added information*

*in the DISCUSSION section***– Lines: 213-224**

- Lines 193-195: Mentions the long interval between sample collection and testing - please clarify. How long? In what way would this interfere with the results?

***ANSWER:*** *Thanks for pointing it out. We clarified this information in the DISCUSSION section-* **Lines: 246-248**

**“***There was a gap between the blood sample collection and the serum analysis, although a proper storage was assured. The number of tested participants was superior to the sample size estimate, but a relevant number of participants from the main PAI study was not included for the current ancillary analysis.”*

The samples were collected in 2017, but serological tests were performed in 2019 in part of the PAI study sample. However, there was no damage to the current study.

Lines 193-200, Limitations: Recommend mentioning potential for serological cross-reaction.

- ***ANSWER:*** *Thanks for pointing it out. We now have added information*

*in the DISCUSSION section***– Line: 244, 245**

```
 “...potential serological cross-reactions could not be addressed.”

Reviewer(s)' Comments to Author:

Reviewer: 1
```

- In line 48 from the Methods section, the authors should provide more explication about the choice of the 30 to 70 years of age range for recruitment.

- ***ANSWER:*** *Thanks for pointing it out. We now have added information*

*in the METHODS section***– Lines: 49-56**

**“**The current analyzes was carried out as an ancillary study of the Project of Atherosclerosis among Indigenous Populations (PAI) study.”

As it is an auxiliary study of the PAI, it complies with the inclusion criteria of the sample from the original initial study.

- In line 48, the Authors should clearly state the approval number from Research Ethics Committee, regarding the work with Traditional Populations.

- ***ANSWER:*** *Thanks for pointing it out. We now have added information*

*in the METHODS section***– Lines: 57-61**

**-**The authors used a commercial kit for detection of anti-chikungunya virus IgG by enzyme-linked immunosorbent assay. Since it is widely known the occurrence of cross-reactions in serological tests to detect arbovirus infections, the authors should provide more details whether other tests were performed to detect other arbovirus species, or at least discussing the performance data from the kit used in the study.

- ***ANSWER:*** *This is a very interesting point. We now have added information in the METHODS section***– Lines: 116-122.**

***“****Although cross-reaction is a challenge in seroprevalence studies for arbovirus, it is particularly problematic in arboviruses of the same family, such as Dengue Fever, Yellow Fever and more recently Zika Fever [22]. Regarding CHIKV, commercial ELISA diagnostic tests were evaluated and showed specificity and sensitivity of 95% and 88% for IgG, respectively, with 18% false positive for IgM ELISA [23]. In addition, the EuroImmun anti VCHIK ELISA IgG assay (EUROIMMUN AG, Lüebeck, Germany) showed 95·4% sensitivity and 100% specificity in validation studies on another specific population, in previous studies.”*

**-** In line 90, the authors should clearly provide the exact number of samples discarded.

- ***ANSWER:*** *We now have added informationin the METHODS section***– Lines: 111-115.**

*“*Serological tests were performed on 451 participants of the PAI study who agreed to be included and had viable serum samples at the time the test became available for the study, during 2019. Only 03 boderline samples from the Fulni-ô group were discarded. Fifteen samples lipemic, icteric, hemolyzed, or failed samples were discarded. No other serological tests were performed for other arboviruses in this study.”

*That is, 18 samples were discarded.*

**Reviewer: 2**

**-** The methodology of this ancillary study is well described; however, it is necessary to state the sampling strategy. Also, I suggest clarifying the term “stratified” in line 44: does it refer to stratification in the context of a complex sampling design of the PAI study or to the three exposure groups?

- ***ANSWER:*** *Thanks for pointing it out. We now have added information*

*in the METHODS section (***Study design and sample selection)– Lines: 46-57.**

The stratification of the groups in this study, follows the same stratification of the PAI study.

-The exposure and control groups were adequately selected in terms not only of ethnicity but also of a gradient of urbanization, which allowed the evaluation of the contrasts of main interest; however, it is not clear the reason(s) behind testing a hypothesis of association between body mass index (BMI) and seroprevalence (lines 40-41): I suggest to provide evidence in support of the biological plausibility and/or clinical relevance of such relationship.

***ANSWER:*** *This is a very interesting point. We now have added information in the METHODS section (***Anthropometric data collection)– Lines: 96-98.**

**“**Obesity has been described as a major risk factor for severe clinical presentations of arbovirosis. [18] Therefore, we included obesity data in our analysis, as measured by BMI, according to the World Health Organization recommendations.”

-The statistical approach seems to be consistent with the aim of the study, however, it is not clear whether the PAI study (and the ancillary) had a complex sampling design and, therefore, whether or not point and uncertainty estimates should account for it: Please provide further details.

***ANSWER:*** *Thanks for raising this important issue. We now have a totally restructured subheading on “Study design and sample selection”, in the first paragraph of the Methods section. Therefore, we expect to have provided additional information that complies with your concerns****.***

**Reviewer #1:**

**-** In table 1, there is a typing error in the "Mean DP", perhaps it might be "SD- standard deviation".

***ANSWER:*** *Thanks for pointing it out. The table error has been corrected: "SD”.*

**Reviewer #2:**

- Results from descriptive statistics and univariate analysis are clear and correspond to the analysis plan, under de assumption that PAI (and the ancillary) study did not followed a complex sampling design (i.e., simple random sampling): Please, consider to change the title of table 1 (“Description of anthropometric parameters (n = 433)”) as it shows more than anthropometric parameters for which there is a high proportion of missing data (22.2%).

***ANSWER:*** *Thanks for pointing it out. We have modified the title of table 1:* “*Description of characteristics of the total sample studied (n = 433)”*

- I have the following concerns about the multivariate analysis: 1. What was the control group? If this group was the “urbanized control community” as its denomination suggests, then is incorrect to present a prevalence ratio for that group (Table 3: APR=0.966), furthermore, an APR lower than the unity (0.608) for the Fulni-ô is not plausible since prevalence in this group is 78.3% (213/272) as compared to 5.8% (3/52) in the “urbanized control community”; 2. Alternatively, if the“control” group was the Truká, then there would be a zero prevalence in the denominator of any PR (or APR); and 3. There is no indication of which variables the prevalence ratio was adjusted for or what were the reasons behind selecting them to be included in a multivariate model (the univariate analysis does not support a relationship to prevalence for any covariate).

***ANSWER:*** *Thanks for pointing it out. We clarified this information in the RESULTS section***.** *After reviewing the statistical data and the variables presented, we conclude that multivariate analysis is not appropriate. Therefore, we portrayed the misconception and agreed with the reviewer.*

*-***Reviewer #1:** The authors claim that the "less urbanized Indigenous community might be the most endangered for new viral outbreaks". This sound a very strong statement, since the authors provide information about only one arbovirus species, and the prevalence of CHIKV infections in the indigenous populations analyzed indicated that this population likely presents herd immunity to CHIKV. The authors should reformulate the sentence.

***ANSWER:*** *Thanks for pointing it out. We clarified this information in the DISCUSSION section-* **Lines: 171- 173.**

**“**Our results suggest that the most traditional less urbanized Indigenous community was the most exposed to CHIKV, demonstrated by the highest serological rate of infection.”

**-** **Reviewer #2**: In general, the validity of a conclusion regarding prevalence is contingent upon the sampling design, something that the authors did not describe in the manuscript and cannot be traced from the references to the main study (PAI). Furthermore, there are concerns about the analysis itself that must be clarified before attempting an interpretation of the results.

***ANSWER:*** *Thanks for pointing it out. We clarified this information in the METHODS section-* **Lines- 51-57 and 124-125:** These lines deal with the sample, with its PAI study stratifications.

Lines- 116-120 (**Statistical Analysis):** These lines describe the sample planning for the PAI study and the ancillary study.

- **Reviewer #1:** The authors recruited patients from indigenous Populations in the São Francisco Valley, Brazil between

August 2016 and June 2017, to collect clinical data and blood samples that underwent serology testing for anti-CHIKV

IgG by ELISA. By analyzing the data from 433 patients, the authors argues that there is a high prevalence of

Chikungunya virus infection in the Fulni-ô indigenous population between 2016 and 2017. The study found 78·3% of the samples from Fulni-ô group tested positive for anti-CHIKV IgG. Authors' conclusions are consistent with the results presented in the study, they provide a discussion about limitations of the study as well as on previously published related literature.

- In line 13 from the introduction section, the authors should provide a reference for the claim: "in the semi-arid region of the Brazilian Northeast (the poorest region in the country) and in the Amazon Forest region. "

***ANSWER:*** *Thanks for pointing it out. We now have added information in the INTRODUCTION section. Line 13: “...*and in the Amazon Forest region [7].”

- **Reviewer #2:** Nicacio et al., conducted a Chikungunya serosurvey in two indigenous groups and in an urbanized control community (30-70 years old) in Northeast Brazil, as an ancillary study of the Project of Atherosclerosis among Indigenous Populations (PAI). This is a relevant research question because of the particularities and vulnerability of the target populations; however, the absence of a detailed description of the methodological approach – specifically, the sampling design – of both the principal and the ancillary studies precludes to reach meaningful inferences regarding the burden of the infection as determined by estimates of seroprevalence.

***ANSWER:*** *Thanks for pointing it out. We clarified this information in the METHODS section -“***Study design and sample selection”- Lines: 46-61 and** **“Statistical Analysis”- Lines: 124-132**

We thank the reviewers for their valuable comments. We have been greatly helped to improve the quality of our research.

Sincerely,

Jandir Mendonça Nicacio
