# Supplementary material for: Anti-chikungunya virus seroprevalence in Indigenous groups in the São Francisco Valley, Brazil
Source: PLoS Negl Trop Dis. 2021 Jun 28;15(6):e0009468. doi: 10.1371/journal.pntd.0009468 (PMC8238182; doi:10.1371/journal.pntd.0009468)
Supplement: Attachment — Submitted filename: Revised Article with Changes H - bmf.docx [file pntd.0009468.s005.docx]

**Anti-chikungunya virus seroprevalence in indigenous groups in the São Francisco Valley, Brazil**

Jandir Mendonça Nicacio^1¶*^, Antônio Ricardo Khouri Cunha^2,3,4, #a¶^ , Antônio Marconi Leandro da Silva^1&, #b^, Manoel Barral Netto^2,3,5,&^, João Augusto Costa Lima^6,&^, Ana Marice Teixeira Ladeia^7,&^, Rodrigo Feliciano do Carmo^8,&^, Anderson da Costa Armstrong^1¶^

^1^ Associate Professor, Medical School, UNIVASF, Petrolina-PE, Brazil.

^2^ Oswaldo Cruz Foundation/Fiocruz, Institute Gonçalo Moniz, Salvador, Brazil

^3^ Federal University of Bahia School of Medicine – UFBA

^4^ Rega Institute for Medical Research, KU Leuven, Leuven, Belgium

^5^ Instituto Nacional de Ciência e Tecnologia de Investigação em Imunologia, São Paulo- SP, Brazil

^6^ Cardiology from Johns Hopkins University

^7^ Postgraduate Course in Medicine and Human Health, Bahiana School of Medicine and Public Health, Salvador, Brazil.

^8^ Collegium of Pharmaceutical Sciences, Universidade Federal do Vale do São Francisco, Petrolina (PE), Brazil.

^#a^ Current Address: Oswaldo Cruz Foundation/Fiocruz, Institute Gonçalo Moniz, Salvador, Brazil

^#b^ Current Address: Department of Medicine, Federal University of Vale Do São Francisco (UNIVASF), Petrolina, Pernambuco, Brazil.

^*^Corresponding author

E-mail: [jdinicacio@gmai.com](mailto:jdinicacio@gmai.com) (JMN)

^¶^ These authors contributed equally to this work.

^&^These authors contributed equally to this work.

**Abstract**

**Background**

Chikungunya fever (CHIKF) is a serious public health problem, with a high rate of infection and chronic disabling manifestations that affected more than 2 million people worldwide since 2005. In spite of this, epidemiological data on vulnerable groups such as Indigenous people are scarce, making it difficult to implement public policies in order to prevent this disease and assist these populations.

**Objective**

We describe the serological and epidemiological profile of chikungunya virus (CHIKV) in two Indigenous populations in Northeast Brazil, and in an urbanized control community and explore associations between CHIKV and anthropometric variables in these populations.

**Methodology/Principal Findings**

We report on Cross-sectional ancillary study of the Project of Atherosclerosis among Indigenous Populations (PAI), that included people 30 to 70 years old, recruited from two Indigenous tribes (less urbanized Fulni-ô and more urbanized Truká people) and a control urbanized non-indigenous group from the same area. Subjects underwent clinical evaluation and were tested for anti-CHIKV IgG by enzyme-linked immunosorbent assay. Serological profile was described according to ethnicity, sex, and age. The study population included 433 individuals distributed in the ethnic groups: 109 (25·2%) Truká, 272 (62·8%) Fulni-ô, and 52 (12%) from the non-Indigenous urbanized control group. Overall prevalence of CHIKV IgG in the study sample was 49·9% (216; 95% CI: 45·1 – 54·7). When the sample was stratified, positive CHIKV IgG was distributed as follows: no individuals in the Truká group, 78·3% (213/272; 95% CI: 72·9 – 83·1) individuals in the Fulni-ô group, and 5·8% (3/52; 95% CI: 12·1 – 16) individuals in the control group.

**Conclusions/Significance**

Positive tests for CHIKV showed a very high prevalence in a traditional Indigenous population compared to the absence of anti-CHIKV serology found in the urbanized Truká people and in the non-indigenous people, although all regions are located in the same area.

**Author summary**

Chikungunya fever, caused by the vector-borne chikungunya virus (CHIKV), is a serious public health problem, with a high rate of infection and disease. CHIKV is a cosmopolitan virus, which has inflicted severe damage in 46 countries in the Americas and responsible for chronic disabling manifestations. In spite of this, epidemiological data on vulnerable groups such as Indigenous people are scarce. We report on a cross-sectional study describing the seroprevalence of CHIKV in Indigenous groups in the São Francisco Valley, Brazil, in association with anthropometric data. The study population included 433 individuals distributed in the following ethnic groups: 109 (25·2%) Truká, 272 (62·8%) Fulni-ô, and 52 (12%) from the non-Indigenous urbanized control group When the sample was stratified, positive CHIKV IgG was distributed as follows: no individuals in the Truká group, 213/272 (78·3%; 95% CI: 72·9 – 83·1) individuals in the Fulni-ô group, and 3/52 (5·8%; 95% CI: 12·1 – 16) individuals in the control group. This study shows, for the first time, that CHIKV circulated in an indigenous population (Fulni-ô) in the São Francisco Valley, in 2016 and 2017. The finding strikingly differs from the absence of anti-CHIKV serology found in the Truká people and in the urban region of Juazeiro, Bahia.

**Keywords:** arbovirosis; chikungunya fever; Fulni-ô; Truká; seroprevalence.

**INTRODUCTION**

The 21st century has been marked by major viral diseases outbreaks that progressed to pandemics, as was the case of the “Swine Flu” in 2009, which caused more than 18,000 deaths; chikungunya, in the early 2000s with rapid spread, reaching global proportions [1]; the Zika epidemic, in 2015, associated with several cases of Guillain-Barré syndrome and congenital microcephaly [2], and finally coronavirus disease 2019 (COVID-19), which has infected more than 5 million people worldwide and is still expanding [3]. In spite of this, little is known regarding the behavior of viral epidemics in different populations, taking ethnic characteristics and access to the health system into account.

Chikungunya fever (CHIKF), which is caused by the chikungunya virus (CHIKV), was silent for three decades until major outbreaks affected people in Kenya and India in 2004 and 2005 [4,5]. In 2013, it affected more than 1·5 million people in Central and South America [6]. In 2014, the disease officially arrived in Brazil, and the first autochthonous cases were registered, almost simultaneously in the semi-arid region of the Brazilian Northeast (the poorest region in the country) and in the Amazon Forest region [7]. Within about two years, nearly one million cases of CHIKF had been registered in the Americas, *Aedes aegypti* being the main vector [8,9]. However, the impact of CHIKV on Brazilian Indigenous populations is still unknown.

In Brazil, there are officially 760,350 Indigenous people, composing 416 ethnic groups and 6,238 villages, who receive health care assistance from 1,199 Indigenous Primary Health Units [9]. Indigenous groups are distributed throughout the vast territory of Brazil, living in various stages of urbanization. The Northeast Region of Brazil, where European colonization began, still has Indigenous peoples with diverse ethnicities and traditions [10]. However, very few studies have evaluated the impact of new epidemics on Indigenous communities [11].

To date, in Brazil, the largest records of probable case reports of CHIKF were in the years 2016 and 2017, with 277,882 and 185,605 cases, respectively, and more than 151,000 confirmed cases during these years. The states in the Northeast Region had the highest incidence rates in 2016 and 2017 (420·3 cases/100,000 inhabitants and 249·5 cases/100,000 inhabitants, respectively) [12]. In 2018, the Northeast Region showed a significant drop in notifications, becoming fourth out of five regions in terms of incidence (19·5 cases/100,000 inhabitants) [13]. In 2020 (up to EW 46), the Northeast registered an incidence of 37·5 cases/100,000 inhabitants, showing an increase in cases compared to the previous year [14].

Currently, the Northeast state of Pernambuco, which registered new CHIKV case rates of 531·4 cases/100,000 inhabitants in 2016, has an incidence of 34 cases/100,000 inhabitants by EW 38 of 2020 [12, 14]. These notifications, however, did not take the vulnerable Indigenous populations of the country into consideration.

Seroprevalence studies on arboviruses in Indigenous groups in the Americas are rare. In 2004, one study identified the seroprevalence of dengue IgG in the Yukpa and Barí Indigenous tribes in Venezuela [15]. Later, in 2015, a seroprevalence study of CHIKV, hantavirus and rickettsia was conducted in the Tuchín Indigenous community in northern Colombia, finding no cases of CHIKV in this population [16].

This study describes the serological and epidemiological profile of CHIKV in two Indigenous populations in the Northeast of Brazil, at different stages of urbanization, in addition to an urbanized control community in the same region. Our hypothesis was that the lower degree of urbanization groups, strongly related to worse sanitation conditions and health education, would have the highest prevalence of CHIKV. Furthermore, it explores the associations between the serological profile of CHIKV and anthropometric variables usually related to worse outcomes in arbovirosis outbreaks [17].

**METHODS**

**Study design and sample selection**

This is a descriptive, cross-sectional, seroprevalence study, with stratified sample according to the degree of urbanization, carried out in specific populations of the São Francisco Valley in Northeast Brazil, composed of Indigenous groups from two tribes (Fulni-ô and Truká people) and a control non-indigenous urbanized group (from Juazeiro city, state of Bahia). The current analysis was carried out as an ancillary study of the Project of Atherosclerosis among Indigenous Populations (PAI) study.

The PAI study protocol has been previously described in detail [18]. In summary, PAI was initiated in 2016 as an observational study, primarily aimed to access cardiovascular health in two indigenous groups with different degrees of urbanization and a control group totally urbanized. The PAI study included individuals of both sexes, 30 to 70 years old, without known cardiovascular disease or renal failure requiring hemodialysis, upon authorization to conduct the study and after signing the informed consent term [18]. The initial phase of the PAI study was ended in 2017, after including a total of 1061 participants (321 from Fulni-ô tribe; 351 from Truká tribe; 389 from Juazeiro city). The study was approved by the National Research Ethics Council (CONEP number 1.488.268), the National Indigenous Foundation (Fundação Nacional do Índio- FUNAI; process number 08620.028965/2015-66), and the indigenous leaders of the participating groups. All participants provided informed consent before taking part in the study [18,19].

**Indigenous Groups**

Three groups were included: one Indigenous tribe characterized by low level of urbanization (Fulni-ô, located on the banks of the Ipanema River in the São Francisco Basin); one tribe already affected by the urbanization process (Truká, whose territory is crossed by the northern axis of the São Francisco River Transposition Project); and a control group located in a city with a totally urbanized area and a low profile of migration in the municipality of Juazeiro, Bahia, also on the banks of the São Francisco River [18].

The Fulni-ô Indigenous tribe is located in the municipalities of Aguas Belas and Itaíba, in the *agreste* region of the state of Pernambuco, and it comprises an area of 12,000 m^2^ with 4,689 people, showing signs of less urbanization. Once a year, the Fulni-ô people are required to be confined in isolation to non-indigenous people for three months in a separate area adjacent to the tribe for the ritual known as the Ouricuri. The Fulni-ô is the only indigenous group in Northeast Brazil to maintain their original language for daily use [10,18].

The Truká group, on the other hand, comprises people located in the semi-arid of the same state, in the middle of the São Francisco River; the population comprises 2,981 people, in an area of 6,000 m^2^, in the municipality of Cabrobó, on the island known as Ilha de Assunção or Ilha Grande, which is part of the archipelago of Asunción showings anthropological signs of greater urbanization [10, 20]. They are known as a rural community. The geographical locations of the Truká and Fulni-ô tribes are shown in the Figure 1.

Both groups are assisted by a family group of health professionals, comprising a physician, a nurse, and a group of assistants and local community workers.

Figure 1. Truká and Fulni-ô tribes geographical locations

**Anthropometric data collection**

Weight, age, height and body mass index (BMI) were collected from August 2016 (epidemiological week EW 34) to June 2017 (EW 22), in two collection periods. During EW 34/2016, only anthropometric data, and biological samples were collected in the Fulni-ô group. In EW 22/2017, a second moment of collecting anthropometric data and biological samples was performed, now not only in the Fulni-ô group, but also in the Truká tribe and in the control group. Data collection followed the criteria of the PAI Study [18]. For analysis, age was stratified as 30 – 40 years, 41 – 50 years, 51 – 60 years, and 61 – 70 years.

Obesity has been described as a major risk factor for severe clinical presentations of arbovirosis. [18] Therefore, we included obesity data in our analysis, as measured by BMI, according to the World Health Organization recommendations. In relation to BMI (kg/m2), individuals were classified in a simplified manner as underweight (< 18-5), overweight (≥ 18-5 and < 25), and obese (> 30) according to the World Health Organization [21].

**Collection of biological material**

Peripheral blood collections were also performed in EW 22/2017, with right or left antecubital fossa venipuncture, removing 5 to 10 ml of venous blood, after appropriate antisepsis and transported in refrigerated boxes at 2 to 8 ºC. Afterwards, serum was centrifuged in 1.5 ml Eppendorf tubes and conserved at −20 to −70 ºC. These samples were stored at the Instituto Gonçalo Moniz, FIOCRUZ Bahia and the Laboratory of Clinical Analysis, LPC, Salvador, Bahia.

**Serological tests**

Serological tests for anti-chikungunya virus IgG by enzyme-linked immunosorbent assay (ELISA) from Euromimmun (Code: EI 293a-9601G) were performed following manufacturer recommendations. Values with relative index ≥ 1·1 were considered positive; values ≥ 0·8 and < 1·1 were considered borderline, and values < 0·8 were considered negative. Serological tests were performed on 451 participants of the PAI study who agreed to be included and had viable serum samples at the time the test became available for the study, during 2019. Only 3 borderline samples from the Fulni-ô group were discarded. Fifteen lipemic, icteric, hemolyzed, or failed samples were discarded. No other serological tests were performed for other arboviruses in this study.

Although cross-reaction is a challenge in seroprevalence studies for arbovirus, it is particularly problematic in arboviruses of the same family, such as dengue fever, yellow fever and more recently Zika fever [22]. Regarding CHIKV, commercial ELISA diagnostic tests were evaluated and showed specificity and sensitivity of 95% and 88% for IgG, respectively, with 18% false positive for IgM ELISA [23]. In addition, the EuroImmun anti VCHIK ELISA IgG assay (EUROIMMUN AG, Lüebeck, Germany) showed 95·4% sensitivity and 100% specificity in validation studies on another specific population, in previous studies. [24]

**Statistical Analysis**

Sample size calculation for the PAI study was previously described [18], computing a total of 957 participants (319 on each of the three groups). For this ancillary study, the computed sample size was a total of 364 participants, considering the reported prevalence of Chikungunya for municipality in the state of Bahia in December 2015 of 45·74% [25,26], using an online tool <http://sampsize.sourceforge.net/iface/>.

The choice of a municipality in Bahia (also located in the Northeast region) as a reference for the prevalence estimated in the sample calculation was due to the absence of seroprevalence data in the regions close to the tribes. In addition, this municipality has climatic and demographic similarities with the cities where the Truká and Fulni-ô groups are located [27].

Data were entered into the SPSS computer program (SPSS Inc., Chicago, IL, USA, Release 16·0·2, 2008) twice, with automatic consistency and amplitude checking. Categorical variables were presented as absolute and relative frequencies, and continuous variables were presented as median (first [1Q] – third [3Q] quartiles) after verification of data normality by the Kolmogorov-Smirnov test. The frequency of infection by CHIKV was described in terms of percentage, and imprecision was quantified by 95% confidence interval (CI). In univariate analysis, positive and negative individuals were compared by Mann-Whitney U test and Pearson’s chi-square (χ^2^) for continuous and categorical variables, respectively.

**RESULTS**

A total of 433 individuals with full available serological and clinical data were included, the majority being young women. Of the total, 118 (35%) individuals were considered obese and 134 (39,8%) were considered overweight (Table 1).

**Table 1.** Description of characteristics of the total sample studied (n = 433)

| **Variables** | | **N** | | **%** | |
| --- | --- | --- | --- | --- | --- |
| ***Sex*** | |  | |  | |
| Male | | 159 | | 36·7 | |
| Female | | 274 | | 63·3 | |
| ***Group*** | |  | |  | |
| Juazeiro | | 52 | | 12·0 | |
| Fulni-ô | | 272 | | 62·8 | |
| Truká | | 109 | | 25·2 | |
| **Age group** | |  | |  | |
| 30 – 40 years old | | 147 | | 33·9 | |
| 41 – 50 years old | | 119 | | 27·5 | |
| 51 – 60 years old | | 114 | | 26·3 | |
| 61 – 70 years old | | 53 | | 12·2 | |
| **Nutritional Status*** | |  | |  | |
| Underweight | | 1 | | 0·3 | |
| Adequate weight | | 84 | | 24·9 | |
| Overweight  Obese | | 134  118 | | 39·8  35 | |
| **Variables** | **Median (1Q – 3Q)** | | **Mean ± SD** | |  |
| Age, years | 47·0 (38·0 – 55·0) | | 47·3 ± 10·7 | |  |
| Weight, kg | 70·3 (63·2 – 83·3) | | 73·6 ± 16·6 | |  |
| Stature, cm | 160·0 (155·0 – 166·0) | | 160·6 ± 8·1 | |  |
| Body mass index, kg/m^2^ | 27·8 (25·1 – 31·6) | | 28·9 ± 0·32 | |  |

LEGEND: SD- standard deviation; 1Q- first quartile; 3Q- third quartile. *96 missing weight and/or

height data.

The prevalence of anti-CHIKV IgG was 49·9% (216/433; 95% CI: 45·1 – 54·7). When evaluated separately by group, 3 (5·8%; 95% CI: 12·1 – 16), 213 (78·3%; 95% CI: 72·9 – 83·1), and 0 (zero) positive individuals were observed in the control, Fulni-ô, and Truká groups, respectively. When we evaluated the Fulni-ô individuals by collection period (EW 34/2016 and EW 22/2017), we observed that the percentage of individuals who were positive for anti-CHIKV IgG was maintained (79·6% and 78·1%, respectively).

When univariate analysis was applied, there was no significant difference between individuals with CHIKV IgG by age group or sex. On the other hand, in relation to the sample groups, the Fulni-ô tribe showed a difference in anti-CHIKV IgG, denoting association in comparison to the control group (P < 0.001). However, it was not possible to make statistical inferences in relation to the Truká Indigenous group due to the absence of positive cases in the sample (Table 2). Similarly, when analysis by age and sex was performed only in the Fulni-ô group, no association was found with anti-CHIKV IgG. (Data not shown)

The association of anti-CHIKV IgG seroprevalence with uncategorized continuous variables (age and BMI) was not statistically significant (Table 2).

**Table 2.** Prevalence of CHIKV IgG antibodies in population groups (Control, Fulni-ô and Truká) and association with age, sex and anthropometric data.

| **Variables** | **Control (n = 52)** | | | **Fulni-ô (n = 272)** | | **Truká (n = 109)** | | **P** |
| --- | --- | --- | --- | --- | --- | --- | --- | --- |
|  |  | **%** | |  | **%** |  | **%** |  |
| **CHIKV IgG** **+** |  | | 5·8 |  | 78·3 |  | 0·0 | <0·001 |
| **Females** |  | | 50·0 |  | 64·3 |  | 67·0 | 0·095 |
| **Age Group** |  | |  |  |  |  |  |  |
| 30 – 40 years |  | | 32·7 |  | 35·7 |  | 30·3 | 0·310 |
| 41 – 50 years |  | | 28·8 |  | 24·3 |  | 34·9 |  |
| 51 – 60 years |  | | 21·2 |  | 27·2 |  | 26·6 |  |
| 61 – 70 years |  | | 17·3 |  | 12·9 |  | 8·3 |  |
| **BMI Group** |  | |  |  |  |  |  |  |
| Underweight |  | | 0·0 |  | 0·6 |  | 0·0 | 0·323 |
| Normal |  | | 24·0 |  | 26·5 |  | 20·6 |  |
| Overweight |  | | 34·0 |  | 42·5 |  | 35·5 |  |
| Obese |  | | 42·0 |  | 30·4 |  | 43·9 |  |
| **Variables** | **Median (1Q - 3Q)** | | | **Median (1Q - 3Q)** | | **Median (1Q - 3Q)** | | **P** |
| Age, years | 47·5 (38·0 - 58·3) | | | 47·0 (37·0 - 55·5) | | 45·0 (39·0 - 53·0) | | 0·587 |
| BMI, kg/m^2^ | 28·8 (25·0 - 32·0) | | | 27·6 (24·6 - 30·7) | | 29·1 (25·7 - 33·0) | | 0·144 |

LEGEND: BMI- body mass index; 1Q- first quartile; 3Q- third quartile.

**DISCUSSION**

Our study shows a high seroprevalence for CHIKV in one traditional Indigenous ethnicity, out of three study groups with different stages of urbanization and diverse environmental conditions. Our results suggest that the most traditional less urbanized Indigenous community was the most exposed to CHIKV, demonstrated by the highest serological rate of infection.

Variable results of anti-CHIKV seropositivity have been found in urbanized areas in Brazil. The study evaluated the prevalence in two cities in the state of Bahia, Feira de Santana and Riachão do Jacuípe, and showed a total seropositivity of 51% in December 2015 [25], whereas in Chapada, a district of Riachão do Jacuípe, a rate of 20% positivity was observed in April 2016 and 11% seropositivity was observed in a community of Salvador, Bahia between November 2016 and January 2017 [28,29]. An epidemiological study evaluating two nearby regions with the same climatic conditions during the same EWs, found different prevalences of 57·1% and 45·7% [25]. This difference in seroprevalence in areas with similar climatic and geographical characteristics is possibly related to several factors, such as population density, degree of vector mosquito infestation, sanitary structure of neighborhoods and commercial activity in the city [10,16,18].

The present report draws attention to the fact that CHIKV seems to have had a particular impact on the Fulni-ô people, the least urbanized Indigenous group in the Northeast Region of Brazil, showing more than 70% seroprevalence, in contrast to the absence of anti-CHIKV IgG in the Truká tribe. This significant and unexpected result in the Fulni-ô tribe differs from most epidemiological studies in the Americas, where estimated and found prevalence was less than 50% [28-30]. To date, few studies have recorded such high prevalences. Among those that have, we highlight 75% in Comoros Island in 2004, 63% in Kenya in 2005 [31,32], and 75·6% in Haiti, in children aged 2 to 14 years, in 2014 [33]. It is, however, important to mention that these studies were conducted in non-Indigenous communities, with different degrees of urbanization.

Interestingly, our findings suggest that the more traditional indigenous tribes might have a very particular epidemiological dynamics, regarding viral outbreaks. In fact, official data indicate a markedly lower prevalence of CHIKV in the non-indigenous areas surrounding the Fulni-ô tribe. The municipality of Aguas Belas has a population of 40,235 people and reported a total 167 suspected cases in 2016, remaining below 12 cases/year in subsequent years (2017-2020). The municipality of Itaíba (total population of 26,256 people), which is adjacent to Aguas Belas, reported 143 cases of CHIKV between the years 2015 to 2017. The non-indigenous municipality raw data was acquired from the official epidemiological vigilance and is available as Supplement material (S1 Appendix and S2 Appendix).

Limitations of the official disease notification in Brazilian municipalities are well known. However, the seroprevalence reported in this study, close to 80% of the evaluated Fulni-ô people, is markedly higher than the seroprevalence in people with suspected disease in the non-indigenous areas adjacent to the Fulni-ô tribe. In 2016, of the 167 cases notified for suspected CHIKV disease in Aguas Belas, 55% were confirmed by laboratory and/or clinical epidemiological criteria. Of the total of 11 cases notified in Itaiba for 2017, only 01 case was confirmed by serological testing (S1 Appendix and S2 Appendix).

Regarding the degree of urbanization in isolated communities, our study also differs from previous assessment of seroprevalence of dengue in the Yukpa (most influenced by urbanization) and Barí Indigenous groups, in the Sierra de Perija- Venezuela, which found higher prevalence in the most urbanized tribe, with greater transit of individuals with the virus within this community, precarious health structure and higher infestation of *Ae. Aegypti* [15].

There is no official data for vector mosquito presence, specific for the studied tribes. Nevertheless, since 2002, the Brazilian Ministry of Health has been conducting a survey in the nearby cities (*Ae. aegypti* Infestation Index Rapid Survey -LIRAa). It consists of a method of monitoring the levels of infestation of *Ae aegypti* larvae in homes, performed by sampling technique, periodically, allowing an entomo-epidemiological survey of the region [33]. In 2017, Aguas Belas showed an *Ae. aegypti* Infestation Index Rapid Survey of 2·0, which is considered “alert,” while in the city of Cabrobó (adjacent to where the Truka tribe is located), this same Index was 0·2, which is considered low. In the same period, the city of Juazeiro (control group) showed an Index of 0·8 (considered low or satisfactory) [34]. The different infestation of the vector mosquito might partially explain the highest prevalence of CHIKV in the Fulni-ô people when comparing to the Truká people. However, it does not explain the impressively higher prevalence of CHIKV in the Fulni-ô people compared to the official data of the surrounding non-indigenous cities.

Another important aspect for our finding of diverse CHIKV prevalence ratios may be the specifics of weather conditions in different regions. Although the tribes are located along the Sao Francisco River Valley, in an area of tropical climate, the Fulni-ô group is located in the *agreste* region, where rainfall is slightly higher compared to the Truká group area, which can favor a greater vector mosquito infestation. [35]. However, it also does not fully explain our results, since other cities with similar weather conditions show variable notifications of suspected CHIKF cases in the same period [36].

There is much discussion about the role of poorly planned urbanization and disorderly growth in the health of the population, especially with regard to contagious or insect borne diseases [37]. The high prevalence of CHIKV in the Fulni-ô people may be favored by the degree of urbanization, with unpaved streets, houses that preserve the original construction, which are partly made of clay, and less sanitary structure [10,18]. The Fulni-ô people also struggle for having adequate water supply, usually storing still water, what might be related to mosquito proliferation. This association of CHIKV with the condition of socioeconomic vulnerability has already been identified in previous studies in the Americas, including in a non-Indigenous community in the Brazilian Northeast [28,38]. These factors, in conjunction with a lifestyle characterized by social coexistence, with strong cultural exchange between families and the preservation of confinement rituals (Ouricuri), have probably influenced the high rate of infectivity and transmissibility, since people live close together with multiple close social interactions, in the same environment of the mosquito vector, making the Fulni-ô Indigenous Reserve an environment conducive to maintaining humans in the viral cycle over the years [10,18].

This study has important limitations that must be considered. In fact, serology was not conducted for other arboviruses prevalent in Brazil and potential serological cross-reactions could not be addressed. There was a gap between the blood sample collection and the serum analysis, although a proper storage was assured. The number of tested participants exceeded the sample size estimate, but a relevant number of participants from the main PAI study was not included for the current ancillary analysis. In addition, there was a lack of clinical data to analyze the association of signs and symptoms with seroprevalence data; thus, we could not address the entire dimension of the clinical relevance related to the chikungunya disease burden in these indigenous populations. It is, however, necessary to highlight that working with closed populations, who are distant from large urban centers and who have distinct social behaviors, makes it difficult to execute projects in this profile, in addition to the intrinsic logistical limitations to approach and data collection in more isolated populations of difficult access.

Chikungunya compromises quality of life, interfering with social and work relationships and often leading to psychic disorders [39,40]. This acutely debilitating febrile illness is a serious public health problem, with the risk of evolving into chronic forms. Indigenous people in the Americas have been through known recurrent infectious diseases outbreaks since colonial times, but little is known on the dynamics of the disease, as well as the long term impact in these populations [41]. It is important to understand the epidemiological dynamics of chikungunya, as well as other new infectious diseases, in traditional vulnerable populations such as Indigenous people for aiding in public health policies directed to the management of chronic forms of the disease and for control of future outbreaks.

In conclusion, our study shows that CHIKV left a heavy burden in a traditional Indigenous population (Fulni-ô) in the São Francisco Valley, with a very high prevalence between 2016 and 2017, regardless age and sex. This finding differs from the low prevalence found in the more urbanized Truká people and in the non-indigenous urban people, although all communities are located in the same region. Understanding the dynamics of epidemics in various locations and their peculiarities in diverse population

groups will allow for more appropriate planning of prevention and containment of future epidemics.

**Acknowledgments**

We would like to thank the Executive Secretariat of Health Surveillance, the members of the team of the Arboviruses Surveillance Management of the State of Pernambuco and V Regional Health Management, Daniela Anastacio and Maria Lucia Xavier for the noticing data in the cities of Aguas Belas and Itaiba. We also thank Acácio Willian Faustino de Andrade for helping us to edit the figure referring to the location map of the Fulni-ô and Truká tribes.

**REFERENCES**

1.	Carissimo G, Ng LFP. Understanding Molecular Pathogenesis with Chikungunya Virus Research Tools. In: Curr Top Microbiol Immunol. Springer, Berlin, Heidelberg.2019. Available from: https://doi.org/10.1007/82_2019_158

2.	Kindhauser MK, Allen T, Frank V, Santhana RS, Dye C. Zika: the origin and spread of a mosquito-borne virus. Bull World Health Organ. 2016;94(9):675-86C. Available from: http://dx.doi.org/10.2471/BLT.16.171082

3.	Coronavirus disease (COVID-19): Situation Report – 130 [Internet]. [place unknown]: World Health Organization; 2020 May 29. Covid-19: Report; [cited 2020 May 4]; Available from: https://www.who.int/docs/default-source/coronaviruse/situation-reports/20200529-covid-19-sitrep-130.pdf?sfvrsn=bf7e7f0c_4

4.	Mackenzie JS, Chua KB, Daniels PW, Eaton BT, Field HE, Hall RA, et al. Emerging viral diseases of Southeast Asia and the Western Pacific. Emerg Infect Dis 2001;7(3 Suppl):497-504. Available from: https://dx.doi.org/10.3201/eid0707.017703

5.	Machado LC, de Morais-Sobral MC, Campos TL, Pereira MR, de Albuquerque M, Gilbert C, et al. Genome sequencing reveals coinfection by multiple chikungunya virus genotypes in a recent outbreak in Brazil. PLoS Negl Trop Dis 2019;13(5):e0007332. Available from: https://doi.org/10.1371/journal.pntd.0007332

6.	Gonzalez-Sanchez JA, Ramirez-Arroyo GF. Chikungunya Virus: History, Geographic Distribution, Clinical Picture, and Treatment. P R Health Sci J 2018;37(4):187-94.

7.	Moizéis RNC, Fernandes TAAM, Guedes PMDM, Pereira HWB, Lanza DCF, Azevedo JWV, et al. Chikungunya fever: a threat to global public health. Pathogens and global health, v. 112, n. 4, 2018 Jun 2018. ISSN 2047-7732. Available from: https://www.ncbi.nlm.nih.gov/pubmed/29806537

8.	Matusali G, Colavita F, Bordi L, et al. Tropism of the Chikungunya Virus. Viruses 2019;11(2). Available from: https://doi.org/10.3390/v11020175

9.	Number of Reported Cases of Chikungunya Fever in the Americas, by Country or Territory 2017 (to week noted): Cumulative cases Epidemiological Week / EW 51 [Internet]. [place unknown]: Pan American Health Organization; World Health Organization; 2027 Dec 22. Chikungunya Fever; [cited 2020 Jun 6]; Available from: https://www.paho.org/hq/dmdocuments/2017/2017-dec-22-phe-CHIKV-cases-ew-51.pdf

10.	Armstrong AC, Ladeia AMT, Marques J, Armstrong DMFO, Silva AML, Morais Junior JC, et al. Urbanização Associa-se com Tendência a Maior Mortalidade Cardiovascular em Populações Indígenas: o Estudo PAI. Arq Bras Cardiol. 2018;110(3):240-5. 2018. Available from: http://www.dx.doi.org/10.5935/abc.20180026

11.	Bolaños A, Montoya-Ruiz C, Perez-Peréz JC, Rodas JD, Mattar S. Seroprevalence of arenavirus and hantavirus in indigenous populations from the Caribbean, Colombia. Rev Soc Bras Med Trop. 2019;53. Available from: https://doi.org/10.1590/0037-8682-0132-2019

12.	Brasil- Ministério da Saúde. Boletim Epidemiológico: Monitoramento dos casos de dengue, febre de chikungunya e febre pelo vírus Zika até a Semana Epidemiológica 50, 2017. 2017; [cited 2020 Jan 15]; Available from: https://portalarquivos2.saude.gov.br/images/pdf/2018/janeiro/10/2017-046-Publicacao.pdf

13.	Brasil- Ministério da Saúde. Boletim Epidemiológico: Monitoramento dos casos de dengue, febre de chikungunya e doença aguda pelo vírus Zika até a Semana Epidemiológica 49 de 2018. 2018; [cited 2020 Jan 15]; Available from: https://portalarquivos2.saude.gov.br/images/pdf/2019/janeiro/02/2018-067.pdf

14.	Brasil- Ministério da Saúde. Boletim Epidemiológico: Monitoramento dos casos de arboviroses urbanas transmitidas pelo Aedes Aegypti (dengue, chikungunya e zika), semanas epidemiológicas 1 a 46, 2020; [cited 2020 Jan 15]; Available from: https://central3.to.gov.br/arquivo/545538/

15.	Valero N, Espina LM, Estévez J, et al. [Immunity to Flavivirus in Amerindian Population of the Sierra De Perijá, Zulia State, Venezuela]. Investigacion clinica 2004;45(4).

16.	Barrera S, Martínez S, Tique-Salleg V, Miranda J, Guzmán C, Mattar S. Seroprevalence of Hantavirus, Rickettsia y Chikungunya in the indigenous population of Tuchín, Córdoba. Infectio 2015;19. Available from: https://doi.org/10.1016/j.infect.2015.02.001

17.	Badawi A, Ryoo SG, Vasileva D, Yaghoubi S. Prevalence of chronic comorbidities in chikungunya: A systematic review and meta-analysis. International journal of infectious diseases: IJID: official publication of the International Society for Infectious Diseases, v. 67, 2018 Feb 2018. ISSN 1878-3511. Available from : https://www.ncbi.nlm.nih.gov/pubmed/29277382

18.	Patriota PVAM. PROJETO ATEROSCLEROSE EM INDÍGENAS (PAI): PROTOCOLO DO ESTUDO E RESULTADOS PRELIMINARES. Petrolina. Dissertação [Mestrado em Ciências da Saúde e Biológicas]-Universidade Federal do Vale do São Francisco; 2019.

19.	Patriota PVAM, Ladeia AMT, Marques J, Khoury R, Barral A, Cruz AA, et al. Echocardiography

and Analysis of Subclinical Cardiovascular Diseases in Indigenous People Living in Different Degrees of

Urbanization: Project of Atherosclerosis Among Indigenous Populations (Pai). Arq Bras Cardiol:

Imagem cardiovasc, v. 33, p. 8, 2020. Available from:

http://departamentos.cardiol.br/dic/publicacoes/revistadic/revista/2020/ingles/Revista04/L3_ARTIGO%

0ORIGINAL_ABC78_Ingles.pdf.

20-	Quadro Geral dos Povos: Povos Indí­genas no Brasil [Internet]. [place unknown]: Povos Indígenas do Brasil. Instituto Sócio Ambienta; 2018. Quadro Geral dos Povos; [cited 2020 Jan 17]; Available from: https://pib.socioambiental.org/pt/Quadro_Geral_dos_Povos

21-	World Health Organization: Body mass index- BMI [Internet]. [place unknown]; 2021. Body Mass Index; [cited 2021 Jan 21]; Available from: https://www.euro.who.int/en/health-topics/disease-prevention/nutrition/a-healthy-lifestyle/body-mass-index-bmi

22.	Alwinn R, Doerr HW, Emmerich P, Schmitz H, Preiser W. Cross-reactivity in flavivirus serology:

new implications of an old finding? **Medical microbiology and immunology,** v. 190, n. 4, 2002 Mar 2002.

ISSN 0300-8584. Available from: https://www.ncbi.nlm.nih.gov/pubmed/12005333

23.	Prat CM, Flusin O, Panella A, Tenebray B, Lanciotti R, Leparc-Goffart

I. Evaluation of commercially available serologic diagnostic tests for chikungunya virus. **Emerging infectious diseases,** v. 20, n. 12, 2014 Dec 2014. ISSN 1080-6059. Disponível em: https://www.ncbi.nlm.nih.gov/pubmed/25418184

24-	Litzba N, Schuffenecker I, Zeller H, Drosten C, Emmerich P, Charrel R, et al. Evaluation of the

first commercial chikungunya virus indirect immunofluorescence test. Journal of virological methods,

v. 149, n. 1, 2008 Apr 2008. ISSN 0166-0934. Available from: https://www.ncbi.nlm.nih.gov/pubmed/18294706

25.	Dias PJ, Costa MN, Campos GS, Paixão ES, Natividade MS, Barreto FS, et al. Seroprevalence of

Chikungunya Virus after Its Emergence in Brazil - Volume 24, Number 4—April 2018 - Emerging

Infectious Diseases journal - CDC 2018. Available from: https://dx.doi.org/10.3201/eid2409.180724

26.	Pourhoseingholi MA,Vahedi M, Rahimzadeh M. Sample size calculation in medical studies.

Gastroenterology and hepatology from bed to bench, v. 6, n. 1, Winter 2013 2013. ISSN 2008-2258.

Available from: https://www.ncbi.nlm.nih.gov/pubmed/24834239

27.	IBGE- Instituto Brasileiro de Geografia e Estatística [Internet]. Brasília; 2020. Cidades e Estados: Riachão do Jacuípe; [cited 2020 Dec 20]; Available from: https://www.ibge.gov.br/cidades-e-estados/ba/riachao-do-jacuipe.html

28.	Cunha RV, Trinta KS, Montalbano CA, Sucupira MV, de Lima MM, Marques E, et al. Seroprevalence of Chikungunya Virus in a Rural Community in Brazil. PLoS neglected tropical diseases 2017;11(1).

29.	Anjos RO. Soroprevalência e fatores associados e infecção prévia pelo vírus chikungunya em um bairro de Salvador. Salvador. Dissertação [Mestrado em Biotecnologia em Saúde e Medicina Investigativa] -Instituto Gonçalo Moniz; 2019.

30.	Slavov SN, Otaguiri KK, Bianquini ML, Bitencourt HTO, Chagas MCM, Guerreiro DSS, et al. Seroprevalence of Chikungunya Virus in Blood Donors From Northern and Southeastern Brazil. Hematology, transfusion and cell therapy 2018;40(4). Available from: https://doi.org/10.1016/j.htct.2018.04.002

31.	Sergon K, Yahaya AH, Brown J, Bedja SA, Mlindasse M, Agata N, et al. Seroprevalence of Chikungunya virus infection on Grande Comore Island, union of the Comoros, 2005. The American Journal of Tropical Medicine and Hygiene, v. 76, n. 6, p. 1189-1193, 2007. ISSN 1476-1645. Available from: https://europepmc.org/article/med/17556634

32.	Sergon K, Njuguna C, Kalani RM, Ofula V, Onyango CO, Konongoi LS, et al. Seroprevalence of Chikungunya virus (CHIKV) infection on Lamu Island, Kenya, October 2004. The American journal of tropical medicine and hygiene. 2008;78(2). Available from: https://doi.org/10.4269/ajtmh.2008.78.333

33.	Poirier MJ, Moss DM, Feeser KR, Streit TG, Chang GJJ, Whitney M, et al. Measuring Haitian children's exposure to chikungunya, dengue and malaria. Bulletin of the World Health Organization 2016;94(11). Available from: http://dx.doi.org/10.2471/BLT.16.173252

34.	LIRAa aponta 357 municípios em situação de Risco para dengue, zika e chikungunya [Internet]. Brasília: Ministério da Saúde; 2017 Nov 28. Lira; [cited 2020 Jun 15]; Available from: https://www.saude.gov.br/noticias/agencia-saude/42041-liraa-aponta-357-municipios-em-situacao-de-risco-para-dengue-zika-e-chikungunya.

35.	APAC- Agência Pernambucana de Águas e Clima [Internet]. Recife-PE: Governo do Estado de Pernambuco; [entre 2017 e 2018]. Monitoramento Pluviométrico; [cited 2020 Aug 30]; Available from: http://www.apac.pe.gov.br/meteorologia/monitoramento-pluvio.php

36.	Informe Epidemiológico Arboviroses: Casos e óbitos de dengue, chikungunya e zika Monitoramento dos índices de infestação [Internet]. Recife: Secretaria Executiva de Vigilância em Saúde; 2017. Casos e óbitos de dengue, chikungunya e zika Monitoramento dos índices de infestação: Período de referência vetorial: SE 03 (01/01/17 a 21/01/17); [cited 2020 Aug 26]; Available from: https://www.mppe.mp.br/mppe/attachments/article/7318/INFORME%20ARBOVIROSES%20SES-PE%20SE%2003-2017.pdf

37.	Almeida LS, Cota ALS, Rodrigues DS. Sanitation, Arboviruses, and Environmental Determinants of Disease: impacts on urban health. **Ciencia & saude coletiva,** v. 25, n. 10, 2020 Oct 2020. ISSN 1678-4561. Available from: https://www.ncbi.nlm.nih.gov/pubmed/32997018

38.	Silva MMO, Ribeiro GdS. Características clínicas e epidemiológicas associadas à infecção por Dengue, Zika e Chikungunya em Salvador, Bahia [Tese on the Internet]. Salvador-BA: Fundação Oswaldo Cruz/ Instituto Gonçalo Moniz; 2018 [cited 2020 Aug 26]. 79 p. Available from: https://www.arca.fiocruz.br/handle/icict/35440 Biotecnologia em Saúde e Medicina Investigativa

39.	Silva NMD, Teixeira RAG, Cardoso CG, Siqueira Junior JB, Coelho GE, Oliveira ESF. Chikungunya surveillance in Brazil: challenges in the context of Public Health. Epidemiol Serv Saude. 2018;27(3):e2017127. Available from: http://orcid.org/0000-0001-8428-3674

40.	Krystosik AR, Curtis A, Buritica P, Ajayakumar J, Squires R, Dávalos D, et al. Community context and sub-neighborhood scale detail to explain dengue, chikungunya and Zika patterns in Cali, Colombia. PLoS One 2017. Available from: https://doi.org/10.1371/journal.pone.0181208

41.	Gurgel CBFM, Almeida EA, Lewinsohn R. Índios, jesuítas e bandeirantes. medicinas e doenças no Brasil dos séculos XVI e XVII [Tese]. Campinas SP: Faculdade de Ciências Médicas da UNICAMP; 2009. 225 p. Doutorado em Clínica Médica.

**S1 Appendix.** **Notification of suspected cases of Chikungunya Fever in Aguas Belas municipality, from 2016 to 2020.**

**S2 Appendix. Notification of suspected cases of Chikungunya Fever in Itaiba municipality, from 2015 to 2017.**
