# Supplementary material for: Anti-chikungunya virus seroprevalence in Indigenous groups in the São Francisco Valley, Brazil
Source: PLoS Negl Trop Dis. 2021 Jun 28;15(6):e0009468. doi: 10.1371/journal.pntd.0009468 (PMC8238182; doi:10.1371/journal.pntd.0009468)
Supplement: Attachment — Submitted filename: Rebuttal Letter Second Revision PLOS.docx [file pntd.0009468.s006.docx]

**REBUTTAL LETTER**

On behalf of all authors, we thank the reviewers for the thorough comments and suggestions on our original manuscript. The points raised were extremely helpful to enhancing our work. Please find in this resubmission a restructured version of our study. In addition, we provide detailed answers to each of your concerns in this rebuttal letter, with correspondent modifications highlighted in the submitted manuscript. We appreciate the opportunity to resubmit our study and hope that the revised paper will be fit for publication in *PLOS NEGLECTED TROPICAL DISEASES*.

Sincerely,

Jandir Mendonça Nicacio, MD

Anderson Armstrong, MD, MSc, PhD

**Comments to the Author:**

The authors made a number of improvements to the manuscript in response to the reviews. However, there are still significant issues to address, as indicated by the reviewer and by my comments below:

- Abstract: The confidence interval for the Fulni-o group does not include the point estimate.

***ANSWER:*** *Thanks for pointing it out. We now have added information in the Abstract and* Author summary *sections (“…and 5·8% (3/52; 95% CI: 1·21 – 16)…”* ).

Abstract and elsewhere: It's not clear what makes one tribe more "urbanized" than another - that term is never defined, and the population density of the two tribes seems similar.

***ANSWER:*** *Thanks for pointing it out. We now have added information of urbanization in the ABSTRAC section* and mainly in the *METHODS* section (**Indigenous Groups)-** Lines 82-90.

*“The definition of urbanization in this study refers not only to population density, soil permeability to rainfall, scarcity of vegetation, and the characteristics of buildings, as defined by the Brazilian Institute of Geography and Statistics (IBGE), but also to the historical and cultural aspects of the indigenous groups [20]. In this context, the Fulniô group has no paved roads, and their houses are more isolated. In addition, they practice an annual three-month period of isolation from non-indigenous peoples, in a ritual known as Ouricuri, and preserve their original language in schools, Yathê, along with Portuguese [17,18]. From this point of view, the Fulni-ô group was considered to be less urbanized. In contrast with the Fulni-ô, the Truká group presents intermediate characteristics of urbanization, with lifestyle changes, and major infrastructural landscape transformation [17,18].”*

```
- Introduction: The first paragraph of the Introduction about pandemics is too generic and does not add to the manuscript -recommend deleting.

	ANSWER: Thanks for pointing it out. We have modified this paragraph, making it less generic and related to the central theme of the manuscript
```

*“The last decades have been marked by arbovirosis outbreaks with impact on public health. Among these, chikungunya fever stands out, in the early 2000s with rapid spread, reaching global proportions [1]. Despite this, little is known about the behavior of this arbovirosis in restricted access groups, considering the ethnic characteristics of each population.”*

```
- Introduction line 10-11: States that South and Central America had 1.5 million chikungunya cases in 2013, but that does not seem to be correct.

	ANSWER: Thanks for pointing it out. We have redrafted the sentence. Lines: 8-9
```

*“In 2013 the disease reaches the Americas, affecting about 50 countries by the end of 2014, with almost 23,000 confirmed autochthonous cases [4,5].”*

- Figure 1: the 'control' area should be shown on the map as well.

**ANSWER**: We have edited the figure to include the control group, as advised.

- Methods, line 102: States that "Peripheral blood collections were also performed in EW 22/2017" which is confusing. Since the section is titled Collection of biological material, all collections should be mentioned here.

**ANSWER**: Thanks for the comment. We reword the sentence. Line: 106

*“Peripheral blood collections were also performed between EW 34/2016 to EW 22/2017...”*

- Methods, lines 125-128: The sample size calculation needs to be explained better. What hypothesis was being tested - was this to compare populations? Estimate the overall prevalence? With what precision? Etc.

**ANSWER**: Thanks for the comment. We reword the sentence. Statistical Analysis section. Lines: 128-134.

*“For this ancillary study, the calculate sample size was a total of 364 participants, considering a population of 7580 indigenous people, 95% confidence interval, 5% precision and an estimated prevalence of Chikungunya virus of 46%, based on a previous prevalence study for a municipality in the state of Bahia in December 2015 [25,26], using an online tool <http://sampsize.sourceforge.net/iface/>. Our hypothesis was that the lower degree of urbanization groups, strongly related to worse sanitation conditions and health education, would have the highest prevalence of CHIKV.”*

- Table 2: This table should show the breakdown of CHIKV seropositivity by variable (eg by age group), not just the overall percentage of participants by age group. So, what percentage of females were seropositive and what percentage of males were seropositive? Also please include confidence intervals.

**ANSWER**: We appreciate the feedback and have constructed a new Table 2 in response to the reviewers' comments. These points are now also highlighted in the Results section and in the Table 2. Lines: 163-169

- Discussion: in multiple places the authors refer to "official data indicate a markedly lower prevalence of CHIKV in the non-indigenous areas" - but no seroprevalence data is shown to compare. Please clarify and provide a reference to the data.

**ANSWER**: Thank you for your comment. We have rephrased the sentence and clarified the scarcity of serological data, in the surrounding municipalities. Due to the absence of official data on seroprevalence in this region, we reformulated the sentence.

- In this revised version of the manucript the authors clarified most of the issues raised before, for example, the reason behind the selection of the control group and the sampling of the ancillary study. Noticebly, the authors acknowledged the error made in estimating prevalence ratios (PR); however, it is not clear to me why didn't they re-estimate the PR (at least for the group with non zero prevalence). I suggest to provide additional information about participation rates by group and if possible, also to include a contrast between the samples of the ancillary (n=433) and main (n=1061) studies. This information might help the authors to account for the non expected results as declared in the discussion section.

**ANSWER**: Dear reviewer, we thank you very much for the guidance. Regarding the multivariate analysis, as suggested, we understand that it is not indicated to apply the adjusted prevalence ratio for all subgroups. However, even considering only the subgroups with positivity for Chikungunya, in the univariate model, all independent variables had P>0.2 (sex, age group, Body mass index). Thus, we did not find it interesting to apply a multivariate model afterwards. However, all unadjusted prevalence rates are described in the results. Lines: 159-168

Regarding the number of participants in each subgroup, we included all viable samples from each subgroup. However, the subgroups were not homogeneous, not with regard to the number of participants in each subgroup. Therefore, we put this aspect as a limitation. Lines:250-254

*“The sample was not homogeneous with respect to the proportion of participants in each subgroup. There was a gap between the blood sample collection and the serum analysis. We assessed all blood samples that were properly stored in a sufficient amount for serological testing. The number of tested participants exceeded the sample size estimate, but a relevant number of participants from the main PAI study was not included for the current ancillary analysis.”*

- The conclusions are supported by the data, however, as I pointed out before (methods), providing further description of the sample that participated in the study might shed light to the interpretation of some unexpected findings, as declared by the authors, or at least to rule out the effect of selection bias due to diferential participation rates across groups.

**ANSWER**: Dear reviewers, thank you for the considerations. In the Methods section (Statistical Analysis; Lines: 128-133), we clarify how the sample size of the target population for the ancillary study was calculated. We clarify that the sample distribution for each subgroup was made according to the availability of viable serum samples.

**Other considerations for reviewers:**

Reflecting on the important contributions by the reviewers, we made a few more editions to meet the requests and make the manuscript more enlightening:

- In the Statistical Analysis session, we clarified the statistical model used. Lines: 140-148

- We include as a limitation the non-homogeneity of the sample in terms of the number of participants in each subgroup. However, all available and suitable samples were included. Lines: 250-254

- To correct the confidence intervals, we reviewed the database and revised the descriptive statistics to ensure once again the veracity of the data.

- Data Requirements:

Please note that, as a condition of publication, PLOS' data policy requires that you make available all data used to draw the conclusions outlined in your manuscript. Data must be deposited in an appropriate

repository, included within the body of the manuscript, or uploaded as supporting information. This includes all numerical values that were used to generate graphs, histograms etc.. For an example see here:

http://www.plosbiology.org/article/info%3Adoi%2F10.1371%2Fjournal.pbio.1001908#s5.

Reproducibility:

To enhance the reproducibility of your results, PLOS recommends that you deposit laboratory protocols in protocols.io, where a protocol can be assigned its own identifier (DOI) such that it can be cited independently in the future. For instructions see [https://journals.plos.org/plosntds/s/submissionguidelines#](https://journals.plos.org/plosntds/s/submissionguidelines)

**Authors:**

Dear editors, we respectfully request a waver to deposit our study data in any foreigner repository, as the Brazilian regulations are very strict regarding sharing data on native indigenous people. If this is seen as necessary for publication, we would like some time to formally request authorization from the proper regulatory institutions (in our experience it might take significant time, though). It might help if you already had a formal authorization from the Brazilian official regulators in previous similar manuscripts and could share it with us. Alternatively, a third-party group of Brazilian researchers with current official authorization to access indigenous data could be named for auditing our data and analysis. In this case, we would supply all data without restrictions. We state, however, that we are responsible for the authenticity of the data and the correctness of the statistical analysis.

We thank the reviewers for their valuable comments. We have been greatly helped to improve the quality of our research.

Sincerely,

Jandir Mendonça Nicacio
