# Supplementary material for: Anti-chikungunya virus seroprevalence in Indigenous groups in the São Francisco Valley, Brazil
Source: PLoS Negl Trop Dis. 2021 Jun 28;15(6):e0009468. doi: 10.1371/journal.pntd.0009468 (PMC8238182; doi:10.1371/journal.pntd.0009468)
Supplement: Attachment — Submitted filename: Rebuttal Letter Second Revision_update PLOS.docx [file pntd.0009468.s008.docx]

**REBUTTAL LETTER**

On behalf of all authors, we thank the reviewers for the thorough comments and suggestions on our original manuscript. The points raised were extremely helpful to enhancing our work. Please find in this resubmission a restructured version of our study. In addition, we provide detailed answers to each of your concerns in this rebuttal letter, with correspondent modifications highlighted in the submitted manuscript. We appreciate the opportunity to resubmit our study and hope that the revised paper will be fit for publication in *PLOS NEGLECTED TROPICAL DISEASES*.

Sincerely,

Jandir Mendonça Nicacio, MD

Anderson Armstrong, MD, MSc, PhD

**Comments to the Author:**

The authors have done a good job of addressing the reviewers concerns, with one major exception: Table 2 still doesnot show the data necessary for this publication. This should be the breakdown of participants and seropositivity, bysubpopulation, by region. For example, it should show of the number of males from the control region, how manywere positive and what percentage. Same for females from the control region, same for males from the indigenousregions, etc; the same breakdown should be shown for other variables such as age, etc. I've tried to demonstrate oneway of displaying this data in track changes in the word document the authors had provided (attached). Also, here areother publications showing the data along the lines of what is needed for this manuscript - it doesn't have to be thisformat exactly, but hopefully will give you the idea of the data needed. Table 1 in this manuscript: https://journals.plos.org/plosntds/article?id=10.1371/journal.pntd.0008355 Table 1 in this manuscript: https://journals.plos.org/plosntds/article?id=10.1371/journal.pntd.0006163

***ANSWER:*** *Thanks for pointing it out. As guidance, we redid table 2, including the percentage of positives in each subpopulation, by variable and following one of the suggested table models.*

Please also clarify what the data at the bottom of Table 2, for age and BMI, represent - is that the mean age and BMIfor positive individuals from those regions? How do those numbers compare with negative individuals from the sameregions?

***ANSWER:*** Dear reviewer, thank you very much for your considerations. The median values referred to the total sample. Reflecting on the suggestions and analyzing the table better, we decided to calculate the medians of age and Body Mass Index for each subpopulation (Control, Fulni-ô and Truká) and compared between the subgroups (the positive only) and within each subgroup (positive and negative), showing no statistical difference for both. Results Section: **Lines-176 to 181.**

To improve the layout of Table 2, we decided to include this information in the text of the manuscript and added a table in the Supporting Information (S1 Table).

Figure Files: While revising your submission, please upload your figure files to the Preflight Analysis and Conversion Engine(PACE) digital diagnostic tool, https://pacev2.apexcovantage.com. PACE helps ensure that figures meet PLOSrequirements. To use PACE, you must first register as a user. Then, login and navigate to the UPLOAD tab, whereyou will find detailed instructions on how to use the tool. If you encounter any issues or have any questions whenusing PACE, please email us at figures@plos.org. Introduction: The first paragraph of the Introduction about pandemics is too generic and does not add to the manuscript -recommend deleting.

**ANSWER**: Thanks for the guidance. We configure figure 1 according to the Preflight Analysis and Conversion Engine (PACE) digital diagnostic tool.

- Data Requirements:

Please note that, as a condition of publication, PLOS' data policy requires that you make available all data used to draw the conclusions outlined in your manuscript. Data must be deposited in an appropriate

repository, included within the body of the manuscript, or uploaded as supporting information. This includes all numerical values that were used to generate graphs, histograms etc.. For an example see here:

http://www.plosbiology.org/article/info%3Adoi%2F10.1371%2Fjournal.pbio.1001908#s5.

Reproducibility:

To enhance the reproducibility of your results, PLOS recommends that you deposit laboratory protocols in protocols.io, where a protocol can be assigned its own identifier (DOI) such that it can be cited independently in the future. For instructions see https://journals.plos.org/plosntds/s/submissionguidelines#

**Authors:**

Dear editors, we respectfully request a waver to deposit our study data in any foreigner repository, as the Brazilian regulations are very strict regarding sharing data on native indigenous people. If this is seen as necessary for publication, we would like some time to formally request authorization from the proper regulatory institutions (in our experience it might take significant time, though). It might help if you already had a formal authorization from the Brazilian official regulators in previous similar manuscripts and could share it with us. Alternatively, a third-party group of Brazilian researchers with current official authorization to access indigenous data could be named for auditing our data and analysis. In this case, we would supply all data without restrictions. We state, however, that we are responsible for the authenticity of the data and the correctness of the statistical analysis.

**Other considerations:**

- Taking advantage of the reanalysis and editing of table 2, we checked the database and the construction of table 1 again, in order to reaffirm and guarantee the integrity of the data and the veracity of the information.
- We review all citations for references throughout the manuscript to ensure that they match.
- - We checked the list of references to set up, according to the PLOS Negletecd Tropical Diseases guidelines and to make sure they follow the citation order.

```
-   We also checked, once again, if there were any retracted
articles.
-	Dear Reviewers, the last reference has been changed to suit the required configuration. However, there was no change in the citation or the content of the manuscript.


We thank the reviewers for their valuable comments. We have been greatly helped to improve the quality of our research.
For us, as an author and researcher, the revisions made were very important for the construction of a consistent manuscript. It was an incredible experience!
```

Sincerely,

Jandir Mendonça Nicacio
